# Pilot-Scale Production of Cellulosic Nanowhiskers With Similar Morphology to Cellulose Nanocrystals

**DOI:** 10.3389/fbioe.2020.565084

**Published:** 2020-09-04

**Authors:** Huihui Wang, Jonathan J. Zhu, Qianli Ma, Umesh P. Agarwal, Roland Gleisner, Richard Reiner, Carlos Baez, J. Y. Zhu

**Affiliations:** ^1^State Key Laboratory of Pulp and Paper Engineering, South China University of Technology, Guangzhou, China; ^2^USDA Forest Service, Forest Products Laboratory, Madison, WI, United States; ^3^Department of Biochemistry, University of Wisconsin-Madison, Madison, WI, United States; ^4^International Center for Bamboo and Rattan, Beijing, China

**Keywords:** cellulose nanocrystals (CNCs), cellulose nanowhiskers (CNWs), cellulose nanofibrils (CNFs), mechanical fibrillation, suspension rheology, carboxylation, dispersion, acid hydrotrope

## Abstract

This study describes a class of cellulosic nanomaterials, cellulosic nanowhiskers (CNWs), and demonstrates scaled-up production with acid recovery using less expensive equipment made of common stainless steel rather than glass-lined steel. CNWs produced using concentrated maleic acid (MA) hydrolysis followed by mechanical fibrillation have morphology similar to MA-produced cellulose nanocrystals (CNCs) and sulfuric-acid-produced CNCs (S-CNCs) but differ in crystallinity. Applications of CNWs as a substitute for CNCs for which morphology and surface charge, rather than crystallinity, are the pertinent characteristics are presented. The tested CNW suspensions have a wider viscosity range of 0.001 to 1000 Pa.s over a variety of shear rates of 0.01 to 1000 1/s compared to S-CNCs of 0.001 to 0.1 Pa.s and are better suited for applications such as rheology modification and 3D printing. This study proposes CNWs as a less expensive and sustainable replacement for CNCs in applications that do not require crystalline properties.

## Introduction

Cellulosic nanomaterials (CNMs) have attracted great interest for a variety of applications due to the renewability of cellulosic raw materials and their unique mechanical and optical properties (Mark, 1967; Revol et al., 1992; Šturcová et al., 2005). To the general scientific community, the term CNMs usually refers to two types of materials: (1) cellulose nanocrystals (CNCs), mainly sulfuric acid CNCs (S-CNCs) produced by concentrated sulfuric acid hydrolysis and dialysis for separation; and (2) cellulose nanofibrils (CNFs), produced by mechanical fibrillation with or without chemical/enzymatic pretreatment of cellulosic fibers. CNCs are highly crystalline, as implied by the name, generally with length of 50–500 nm, or degree of polymerization (DP) of 100–300 (Battista, 1950; Battista et al., 1956; Nishiyama et al., 2003), and diameter of 3–10 nm. However, the range of crystallinity for a material to be classified as CNCs has not been established in literature.

Difficulties in accurately measuring CNC crystallinity using traditional x-ray diffraction technique (Segal et al., 1959; Park et al., 2010) have added more ambiguity to the definition of CNCs. Further compounding the problem is the fact that existing crystallinity measurement techniques, including x-ray diffraction (Segal et al., 1959), NMR (Larsson et al., 1997) and Raman (Agarwal et al., 2010), are ensemble measurements over a volume of cellulose sample and therefore spatially averaged rather than resolved to the cellulose nanofibrils/nanocrystals level. This has led to erroneous references to morphologically CNC-like cellulosic whiskers without high crystallinity as being CNCs (Montanari et al., 2005; Hirota et al., 2010; Leung et al., 2011; Anderson et al., 2014).

We suggest that differentiating morphological dimensions from crystallinity is highly important. CNCs have been used for many applications where high crystallinity is not important, such as rheology modifiers (Ojala et al., 2016; Grishkewich et al., 2017; Molnes et al., 2018), hydrogels (Hou et al., 2017; Kim et al., 2017; De France et al., 2019) and 3D printing (Jia et al., 2017; Palaganas et al., 2017; Hausmann et al., 2018; Sultan and Mathew, 2018; Wang et al., 2018; Li et al., 2019) for which dispersibility and proper morphology, not crystallinity, are the keys to performance. This has significant commercial implications because the cost of producing CNCs is often higher than for other CNMs. Furthermore, difficulties in economic recovery of mineral acids (mainly sulfuric acid) and the need for salt disposal pose significant challenges. Current utilization of the expensive S-CNCs for applications that do not require crystallinity is primarily due to (1) ready market availability, because several S-CNC pilot facilities and a commercial facility have been established, and (2) lack of recognition of the diversity of CNMs, as reflected by the generalization of CNMs as either CNCs (individually separated crystalline particles) or CNFs.

Here we discuss an “in-between,” that is, individually separated, charged, and dispersible CNMs with morphology similar to CNCs but without the specification of crystallinity, which allows flexibility in processing to achieve sustainable and economic production. We call this class of CNMs cellulosic nanowhiskers (CNWs), because the term, though not clearly defined, has been previously used (Braun and Dorgan, 2009; Wang et al., 2012a). Individually separated nanoscale cellulosic whiskers were observed in TEM images of substantially fibrillated cellulose fibers (Wang et al., 2012a). Nanoscale cellulosic whiskers were also obtained from mechanical fibrillation of the cellulosic solid residue (CSR) after concentrated sulfuric acid hydrolysis of bleached fibers (Wang et al., 2012b). They have a similar morphology to CNCs but with low crystallinity due to mechanical fibrillation as measured by x-ray diffraction (Wang et al., 2013). Therefore, they are not CNCs.

Previously, we demonstrated integrated production of both CNCs and CNFs at lab-scale (i.e., gram scale) with desired morphologies using dicarboxylic acids (Chen et al., 2016; Wang et al., 2017). Dicarboxylic acids are weak acids resulting in low CNC yields, but the hydrolyzed CSR can be fibrillated into CNMs for substantially higher overall solids (or CNM) yield. Moreover, as weak solid acids with low solubility dicarboxylic acids have substantial advantages over conventional strong mineral acids in terms of acid recovery (Cai et al., 2020a,b) and lower corrosiveness. Furthermore, maleic acid is a U.S. Food and Drug Administration (FDA)-approved indirect food additive per Code of Federal Regulations 21CFR175-177, and therefore with no environmental impact.

The novelty of the present work is to demonstrate pilot-scale production and characterization of alternative CNMs, i.e., CNWs, as a viable substitute for the crystalline but expensive CNCs, with the objective of sustainable processing at higher yields, better use, and broader commercialization of CNMs. Two different acids were chosen to investigate the scalability of each, as well as different starting materials to produce both lignin free CNWs and lignin-containing CNMs (LCNMs).

## Materials and Methods

### Materials

Bleached kraft eucalyptus dry lap pulp (BEP) from Fibria (Aracruz, Brazil) was used for producing lignin-free CNMs. As described elsewhere (Chen et al., 2016; Zhou et al., 2019), BEP was first soaked in distilled water for 24 h and then disintegrated at 10% solid consistency in a lab disintegrator (TMI, Ronkonkoma, NY, United States) at room temperature for 10,000 revolutions at 312 rpm. Disintegrated pulp was collected after vacuum filtration.

Medium density fiberboard (MDF) fibers were used to produce lignin-containing CNMs. MDF fibers were produced by disk-refining birch (*B. papyrifera*) wood chips in a 30.5-cm pressurized disk refiner (Sprout-Bauer, model 1210P, Muncy, PA, United States), as described previously (Bian et al., 2017b). Birch wood logs were from a tree that was approximately 35 years old harvested from the Rhinelander Experimental Forest, USDA Forest Service Northern Research Station, Rhinelander, WI, United States. After hand debarking and chipping at the USDA Forest Products Laboratory, Madison, WI, United States, wood chips were pre-steamed at 165°C for 10 min with a steam pressure of 0.72 MPa before being fed to the disk refiner at approximately 1 kg (OD weight)/min. Resulting pulp fibers were stored in a plastic bag in a refrigerator for further processing.

Anhydrous maleic acid (MA) and *p*-toluenesulfonic acid (*p*-TsOH), both ACS reagent grade, were purchased from Sigma-Aldrich (St. Louis, MO, United States). A commercial endoglucanase, FiberCare^®^, was complimentarily provided by Novozymes North America (Franklinton, NC, United States). Protein content of FiberCare^®^ was assayed at 7.53 mg/mL.

### Scaled-Up Production of CNMs

**FIGURE 1:**
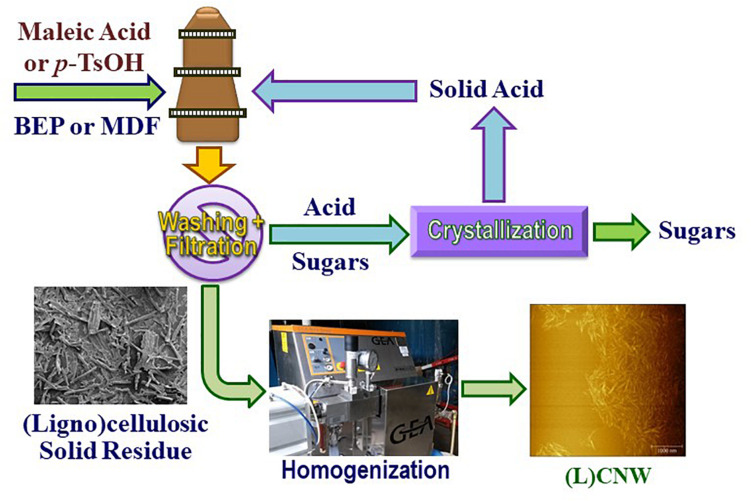
Schematic process flow diagram for pilot-scale production of (ligno)cellulosic nanowhiskers (L)CNWs without separating CNCs (integrated process).

Preliminary small-scale fractionations using 10 g MDF and *p*-TsOH were conducted under a wide range of conditions to obtain predictive delignification information for process scale-up. In the present study, 750 g batches of BEP and MDF fibers were hydrolyzed using concentrated aqueous MA and *p*-TsOH, respectively, following a scheme depicted in Figure 1. MA was applied to BEP fibers and the stronger acid *p*-TsOH was chosen for the MDF fibers. The different hydrolysis conditions were represented by M*xx*T*yy*t*zz* for BEP and P*xx*T*yy*t*zz* for MDF, where M*xx* or P*xx* denote MA or *p*-TsOH acid concentration in *xx* wt%, T*yy* and t*zz* denote hydrolysis temperature in *yy* degree Celsius and reaction duration in *zz* min. All hydrolysis reactions were carried out in a 21-L rotating wood pulping digester at a solid-to-liquor mass ratio of 1:10. Heat-up to the target hydrolysis temperature was only a few minutes. At the end of the reaction period, solids were separated by screen filtration, thoroughly washed, and then fed into a pilot-scale homogenizer (GEA Noro Soavi Ariete NS3015, GEA) for nanoscale fibrillation. The number of passes was varied so as to vary the degree of fibrillation.

### Analytical Methods

Chemical composition of untreated and pretreated BEP fibers was analyzed as described previously (Luo et al., 2010) using the standard two-step acid hydrolysis procedure, with resultant hydrolyzates analyzed by ion chromatography using pulsed amperometric detection (ICS-5000, Dionex, Sunnyvale, CA, United States).

### Characterizations of CNMs

*Crystallinity:* For cellulose crystallinity estimation by Raman spectroscopy, the 380-Raman method (Agarwal et al., 2010, 2013) was used. The method determines the crystallinity of a cellulosic sample based on the ratio of Raman scattering intensity at 380 cm^–1^ over the intensity at 1096 cm^–1^ through calibration using the crystallinity data from wide angle x-ray scattering (WAXS) measurements of a set of well-defined cellulosic samples. CNM samples were analyzed in triplicate using a Bruker MultiRam spectrometer (Bruker Instruments Inc., Billerica, MA, United States) equipped with a 1064-nm 1000-mW continuous wave (CW) diode-pumped Nd:YAG laser operated at 600 mW for sample excitation. Sample pellets were made from ∼0.1 g, and 2048 scans were co-added. Bruker OPUS 7.2 software was used to determine peak positions and process the spectral data. Some samples produced too much fluorescence, so their 380-Raman crystallinity could not be determined. Wide-angle X-ray diffraction measurements of crystallinity were carried out using a Bruker D8 Discover system with Cu-Kα radiation (Bruker Corp., Billerica, MA, United States) at the Material Science Center, University of Wisconsin (Madison, WI, United States). As described previously (Chen et al., 2015), CNM suspensions were first freeze-dried to avoid film formation and to minimize the potential for preferred orientation in the samples. The freeze-dried samples were pressed into 100-mg, 8-mm-diameter pellets using hydraulic compression (180 MPa). A spot size of 0.5 mm was used. Scattering signals were collected in a 2-min period for every specimen. CrI was calculated according to the Segal method (Segal et al., 1959): *CrI* = 100 × (*I*_200_−*I*_am_)/*I*_200_ with *I*_*am*_ the intensity at 2θ = 18°.

#### Atomic Force Microscopy (AFM)

The morphology of CNM samples was characterized by AFM (AFM Workshop, Signal Hill, CA, United States) using a tip curvature radius and vibration frequency of 10–15 nm and 160–225 kHz, respectively. Approximately 0.1wt% suspensions were dispersed with sonication, then a drop of each suspension was placed onto a clean mica surface and air-dried overnight at room temperature prior to characterization.

#### Thermogravimetric Analyses (TGA)

The thermal stability of CNM samples was analyzed using a Pyris 1 TGA (Perkin-Elmer, Inc., Waltham, MA, United States). Samples were dried overnight at 105°C. Aliquots of approximately 8–10 mg were heated from ambient temperature to 700°C at a rate of 20°C/min, with a highly purified nitrogen stream at 20 mL/min continuously passing into the furnace during pyrolysis.

#### Surface Properties

The carboxyl group content of CNM samples was determined by the back-titration method (Jeon et al., 1999; Stojanović et al., 2005). Briefly, 50 mg of CNF suspension (0.2 wt%) was carefully added to 10 mL of 0.1 M NaOH maintained at 50°C and stirred for 30 min. The excess NaOH was back-titrated with standard 0.025 M HCl using phenolphthalein as the indicator. Surface charge was measured using a zeta potential analyzer (Nanobrook Omni, Brookhaven Instruments, Holtsville, NY, United States) based on monitoring electrophoretic mobility using phase analysis light scattering (PALS). The same instrument was used for DLS particle sizing. Each sample was circulated five times for a total of 10 min to obtain averaged size. These two methods were developed for spherical particles but were used as an approximation for CNWs in this study.

#### Suspension Rheology

Rheological analyses of CNC, CNW, and CNF suspensions were performed using a rotational rheometer (MCR 302 Anton Paar Physica) with 43-mm-diameter steel plates in parallel plate geometry. Steady-state shear viscosity curves were generated for all samples in a shear rate range of 0.01 to 1000 s^–1^. The time required to reach steady state at 0.01 s^–1^ was determined by a transient test, and the sampling time used to generate the flow curves was decreased with increasing shear rate. The average of duplicate measurements is reported.

## Results and Discussion

### Process Scale-up

The combined cellulose hydrolysis factor (CHF_G_, where G denotes glucan or cellulose) and combined delignification factor (CDF) [Eqs. (S1a) and (S2a)] developed in our earlier studies (Zhu et al., 2012, 2019; Wang et al., 2017) were used to scale-up (750 g OD weight) experiments, rather than using the individual hydrolysis process parameters such as acid concentration, temperature, and time. This is because cellulose depolymerization, predicted by CHF_G_ (Eq. S1b; Supplementary Table S1 and Supplementary Figure S1), and delignification, predicted by CDF (Eq. S2b; Supplementary Table S1 and Supplementary Figure S2), are considered critical variables in nanoscale fibrillation of bleached chemicals pulps (Qin et al., 2016; Wang et al., 2017) and raw lignocelluloses (Bian et al., 2017b), respectively. Specifically, we used similar reaction severity (i.e., CHF_G_, for BEP or CDF for MDF) in the scale-up as was found in the lab scale experiments to produce the desired CNM morphology. For example, our earlier lab-scale study (Wang et al., 2017) indicated that cellulose DP of approximately 250 or lower is required to obtain short, individually separated CNWs from MA-hydrolyzed BEP through mechanical fibrillation. Therefore, pilot-scale runs M1 and M2 as listed in Table 1 were targeted for CNWs with cellulose DP between 200 and 250; runs M3 and M4 were for producing long fibril-network CNFs. For producing lignin-containing CNWs (LCNWs) using *p*-TsOH hydrolysis of MDF, Run P1 (Table 1) was targeted for 50% delignification; P2 with a lower severity was to explore a lower limit for producing LCNWs. P3 and P4 were for lignin-containing CNFs (LCNFs) with high lignin content and varied morphologies (Supplementary Figure S3).

**TABLE 1:** Reaction severity of scale-up runs, predicted final cellulose DP (BEP), residual xylan X_R_ (BEP and MDF) and residual lignin L_R_ (MDF).

				**Predicted**					**Measured^5^**			
				**CHF_G_;**			**X_R_^3^**	**L_R_^4^**			**Lignin**	**Solids**
**Run**	**Condition^1^**	**Acid**	**Products**	**CDF**	**CHF_X_**	**DP^2^**	**(%)**	**(%)**	**Glucan (%)**	**Xylan (%)**	**(%)**	**yield (%)**
BEP						1027			; 78.1±1.0	; 15.5±0.6	; 0.9±0.5	100
M1	M70T120t120	MA	CNW	37.4;	233	200	1		; 75.5±2.9	27; 7.1±0.4	; 0.9±0.01	58.2
M2	M70T100t120	MA	CNW;CNC	3.58;	22.3	223	45		; 77.7±2.4	57; 10.7±0.7		82.8
M3	M50T90t60	MA	CNF	0.28;	0.8	819	81		; 74.6±4.4	90; 14.6±0.5		95.2
M4	M40T80t60	MA	CNF	0.05;	0.1	976	97		; 63.9±3.1	97; 15.2±1.2	; 0.8±0.02	98.5
MDF									; 34.6±1.0	; 20.8±0.5	; 23.8±0.1	100
P1	P60T80t60	p-TsOH	LCNW	; 616	171		26	34	; 56.6±0.1	36; 12.7±0.2	24; 9.6±1.1	58.4
P2	P50T81t27	p-TsOH	LCNW	; 105	34.6		40	49	; 58.2±0.3	34; 10.8±0.1	23; 8.5±0.7	65.4
P3	P40T80t60	p-TsOH	LCNF	; 73.5	30.1		33	54	; 52.3±3.5	37; 10.9±0.01	55; 18.8±0.5	70.0
P4	P50T73t34	p-TsOH	LCNF	; 62.2	25.2		45	58	; 57.7±1.3	44; 14.7±0.5	56; 21.1±0.4	62.8

*^1^Sample labeling: M and P denote MA and p-toluenesulfonic acid, respectively; *xx* is acid concentration in wt%; T*yy* denotes reaction temperature in *yy* degree C; t*zz* denotes reaction duration in *zz* min. ^2^Eq. S1b; ^3^Eq. S1d; ^4^Eq. S2b. ^5^The numbers before the semi-columns are percent of xylan or lignin retained in water insoluble solids (WISs) calculated from measured yields and compositional analysis of the WISs; numbers after semi-columns are measured component content in the fractionated water insoluble solids.*

We observed that increasing reaction temperature from 100°C (M2) to 120°C (M1), or *CHF*_G_ from 3.6 (M2) to 37 (M1) (an order of magnitude), resulted in only a small reduction in DP from 223 (M2) to 200 (M1) due to the asymptotic nature of reaching LODP; however, xylan retained on the hydrolyzed solids was reduced from 57% to 27% which should facilitate fibrillation.

It is important to note that either *p*-TsOH or MA can be used on bleached fibers for producing CNMs (Chen et al., 2016) or on unbleached fibers for producing LCNMs (Bian et al., 2017a); however, MA cannot delignify the unbleached fibers probably due to extensive lignin condensation, though it is highly effective in solubilizing lignin in untreated wood for producing LCNMs, as recently demonstrated (Cai et al., 2020a,b).

### CNM Morphologies

**FIGURE 2:**
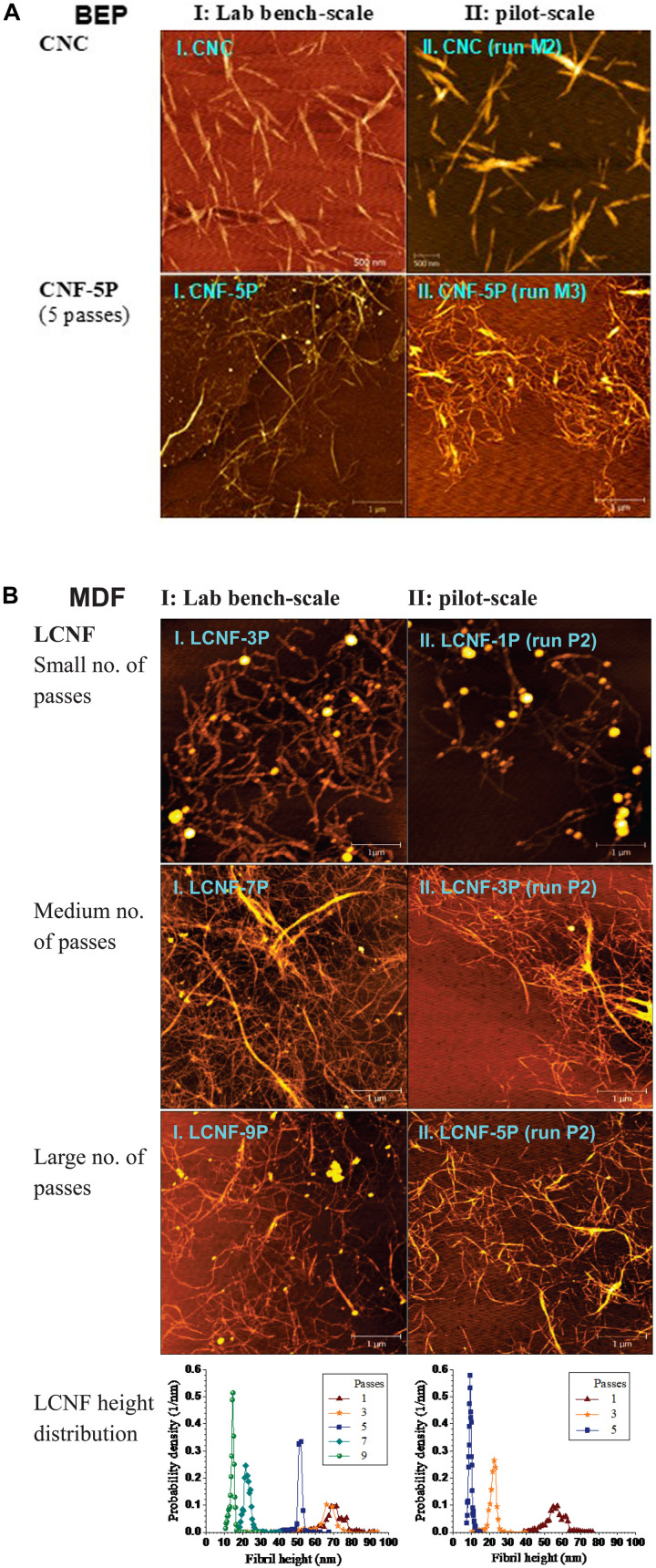
Morphological comparisons of CNMs between samples from lab bench-scale (5 g oven dry weight) runs (I, left panel) and corresponding pilot-scale (750 g) runs (II, right panel). **(A)**: CNC and CNF from BEP using MA; I.CNC: CHF_G_ = 4.08, I.CNF: CHF_G_ = 0.29, 5 passes; II.CNC: CHF_G_ = 3.58, II.CNF: CHF_G_ = 0.28, 5 passes. **(B)**: LCNF from MDF using p-TsOH with varying numbers of passes through fibrillation. I.LCNF: CDF = 70.8, 3–9 passes, II.LCNF: CDF = 105, 1–5 passes. Detailed reaction conditions are listed in Table 1.

Morphology of CNMs from scaled-up runs (750 g) was evaluated by comparing AFM images to those from our earlier published lab-scale (5 g) runs (Wang et al., 2017). Pilot-scale MA-CNCs using severity CHF_G_ = 3.58 (run M2) were similar to, lab-scale severity CHF_G_ = 4.08 demonstrating a successful scale-up, as shown in Figure 2A where I and II designate lab- and pilot-scale, respectively.

CNF production was accomplished by mechanical fibrillation of water-insoluble cellulosic solids from MA hydrolysis of BEP fibers. Fibrillation was carried out using a homogenizer for the pilot-scale and a microfluidizer for the lab-scale run for varied passes or extent of fibrillation. Pilot-scale CNFs from severity CHF_G_ = 0.28 (run M3) were also morphologically similar to lab-scale CNFs from severity CHF_G_ = 0.29 despite differences in their individual hydrolysis conditions, i.e., acid concentration, temperature and reaction time, for example, comparing I.CNF-5P with II.CNF-5P shown in Figure 2A (5P stands for 5 passes in fibrillation).

LCNFs from *p*-TsOH fractionation of MDF were also compared. The higher severity CDF = 105 of the pilot-scale run (P2) than the lab-scale run CDF = 70.8 was due to a slight overshoot in temperature (Supplementary Figure S4) and a delay in quenching the pilot-scale reaction. As a result, pilot-scale LCNFs had a lignin content of 8.5% compared with 16% from the lab-scale run (Bian et al., 2017b). However, morphological similarity between these two runs was achieved simply by applying fewer passes in the homogenizer for the pilot-scale sample, as shown by the AFM images and AFM-measured height distributions (Figure 2B).

**FIGURE 3:**
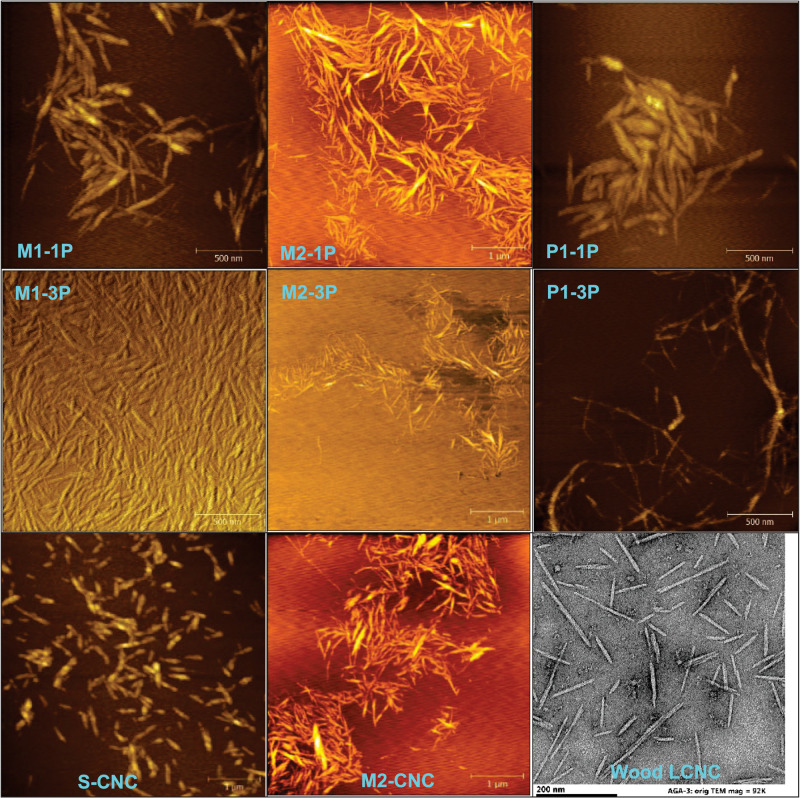
AFM images of CNWs from three high severity pilot-scale runs (M1, M2, and P1) with varied passes (-xP) of homogenization (top and middle rows), S-CNCs (bottom left), CNCs from maleic acid hydrolysis (bottom middle) and LCNCs from sulfuric acid hydrolysis of poplar wood (bottom right).

After accomplishing scale-up of CNCs, CNFs, and LCNFs, we targeted the primary interest of the present study: CNWs – for higher yields with similar morphology to the more crystalline but lower yield CNCs. Figure 3 shows AFM images of (L)CNWs from three high-severity runs: M1, M2, and P1. MA hydrolysis of BEP with subsequent mechanical fibrillation produced individually separated, short whiskers, i.e., CNWs, ranging from 100 to 800 nm in length, with only a single pass through homogenization. Continued fibrillation further reduced aggregations and diameters of the whiskers, resulting in more uniform dimensions. Short and individually separated, lignin-containing LCNWs were obtained after one pass homogenization of the fractionated MDF under P1. However, a sample aliquot after three passes showed significantly longer fibrils, which strongly suggests that P1 condition is not severe enough, or lacking sufficient cellulose depolymerization, to reduce all the fibrils into LCNWs, which will be the topic of future study.

The morphology of pilot-scale CNWs was compared to S-CNCs and CNCs from MA hydrolysis as shown in Figure 3. CNWs from the most severe condition (M1-3P) resembled the S-CNCs except longer, i.e., 365 vs.185 nm and aspect ratio 12.2 vs. 7.7 (Supplementary Table S2), respectively; CNWs from less severe conditions (M2-1P) were very similar to the MA-CNCs separated from this run by dialysis, i.e., 325 vs. 240 nm and aspect ratio 11.6 vs. 9.2 nm (Supplementary Table S2). The pilot-scale LCNWs (P1-1P) were both longer and thicker than LCNCs obtained from direct concentrated sulfuric acid hydrolysis of Wiley-milled poplar wood (Agarwal et al., 2018), i.e., 430 vs. 125 nm and 40 vs. 9 nm (Supplementary Table S2).

### Post-fibrillation Enzymatic Treatment

**FIGURE 4:**
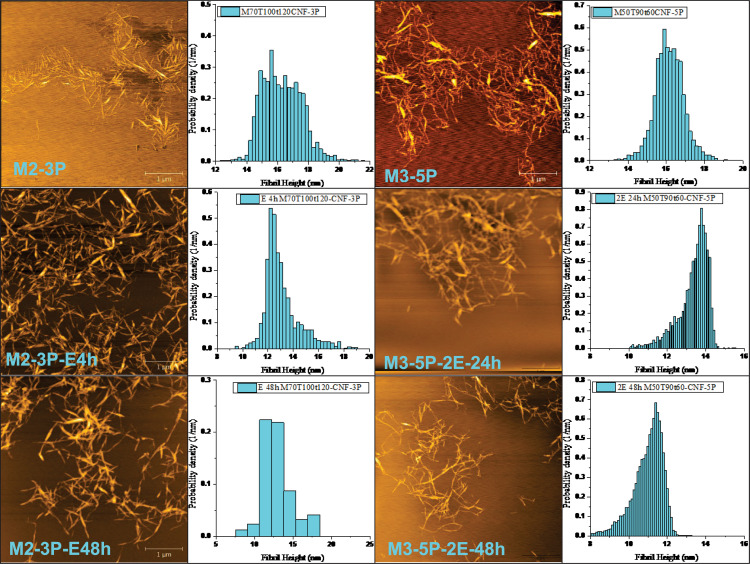
CNM morphological changes through post-fibrillation endoglucanase treatments. FiberCare loading in mg protein/g CNMs: E = 0.2 and 2E = 0.4 for sample M2-3P and M3-5P, respectively. Treatment time: 4 – 48 h.

Post-fibrillation enzymatic treatment can be an effective way to reduce fibril aggregations for producing CNWs with low environmental impact. Two fibrillated CNM samples (M2-3P and M3-5P) were enzymatically treated using a commercial endoglucanase, FiberCare^®^, at very low loadings of 0.2 and 0.4 mg protein/g CNFs, respectively. The results (Figure 4) clearly indicate the effectiveness of endoglucanase treatments to reduce aggregation, diameter, entanglement and length of CNMs. At a lower endoglucanase loading of 0.2 mg/g, M2-3P became much more uniform in diameter with only 4 h of enzymatic treatment (based on AFM measured topographic height distribution, comparing M2-3P with M2-3P-E4h). The average AFM measured fibril height was also reduced from approximately 16 nm to 13 nm. Extended treatment to 48 h (M2-3P-E48h) did not substantially impact the morphology, suggesting potential accessible sites, such as dislocations and disorder regions, have been effectively digested during the shorter period treatment which left fairly uniform and ordered CNW.

Similar effectiveness was also observed for M3-5P with a fibril network morphology but with a similar height of approximately 16 nm (3rd and 4th columns in Figure 4). With a double endoglucanase loading of 0.4 mg/g, the sample network was effectively broken down after 48 h treatment (M3-5P-2E48h). The CNM average height was reduced to approximately 13 and 11 nm after 24 and 48 h treatment, respectively. These results suggest that post-fibrillation enzymatic treatment can be an effective tool to modify CNM morphologies for producing individually separated CNWs, or substantially less entangled CNFs, in agreement with literature (Henriksson et al., 2007; Pääkko et al., 2007; Zhu et al., 2011; Wang et al., 2015, 2016; Yarbrough et al., 2017).

### Crystallinity

Crystallinity index (CrI) of selected samples was measured by both Raman spectroscopy (380-Raman) and X-ray diffraction (Segal methods I and II, with and without background subtraction, respectively) as listed in Table 2. Segal II CrI for M1-1P (77.6%) and M1-3P (74.0%) are lower than that reported for CNCs (81%) produced from concentrated oxalic acid hydrolysis of the same BEP (Chen et al., 2016), but equivalent to that reported for S-CNCs from the same BEP (Chen et al., 2015). In this sense, the high-severity (M1) product could be called CNCs, though not a product of strong acid hydrolysis and dialysis. In contrast to the insensitivity of the Segal method to changes in crystallinity, the 380-Raman method indicated a much larger reduction in CrI (18% vs. 5%) upon homogenization, thereby suggesting these materials should be called CNWs rather than CNCs.

**TABLE 2:** Effect of mechanical fibrillation levels on CrI of CNMs.

		**Segal-I**		**Segal-II**		**380-Raman**	
**Run #**	**Hydrolysis condition**	**1P**	**3P**	**1P**	**3P**	**1P**	**3P**
M1	M70T120t120	89.1	85.1	77.6	74.0	51.7 ± 2.0	42.5 ± 1.0
M4	M40T80t60	85.0	84.3	72.9	72.3	47.6 ± 1.4	42.1 ± 1.0
P1	P60T80t60	82.0	81.6	68.1	67.0	NA*	NA*
P3	P40T80t60	81.8	77.8	67.5	64.2	NA*	NA*

*Not available; could not be estimated due to fluorescence signal in the Raman spectra.

**FIGURE 5:**
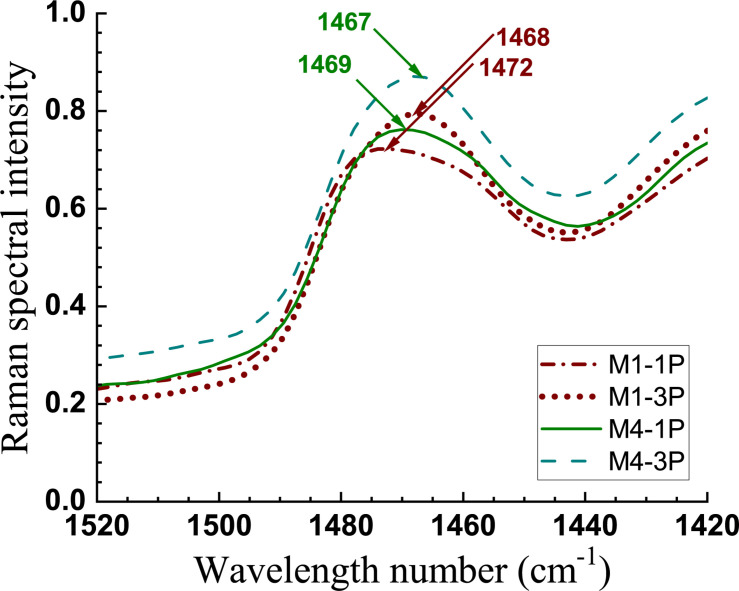
Raman spectra of two sets of CNMs showing the CH_2_ band shifts with increased mechanical fibrillation (1 pass to 3 passes homogenization).

The reduction of crystallinity by extensive mechanical fibrillation was also confirmed from Raman measurements. Raman CH_2_ bands at 1481 and 1462 cm^–1^ have been attributed to crystalline and disordered celluloses, respectively (Schenzel et al., 2005). As shown in Figure 5, the CH_2_ band shifted from 1472 to 1468 cm^–1^ in M1 going from 1 to 3 pass homogenization (i.e., M1-1P to M1-3P). A similar but smaller shift (1469 to 1467 cm^–1^) was observed in samples from less severe hydrolysis (i.e., M4-1P to M4-3P).

Another confounding factor with CNM classification via crystallinity measurement is illustrated with the less severity samples P1 and P3, where unlike the M1 sample with low xylan and near-zero lignin content (Table 1), the presence of hemicelluloses and/or lignin obscures the reduction of CrI after more homogenization, as shown in Table 2.

### Thermal Stability Analyses

**FIGURE 6:**
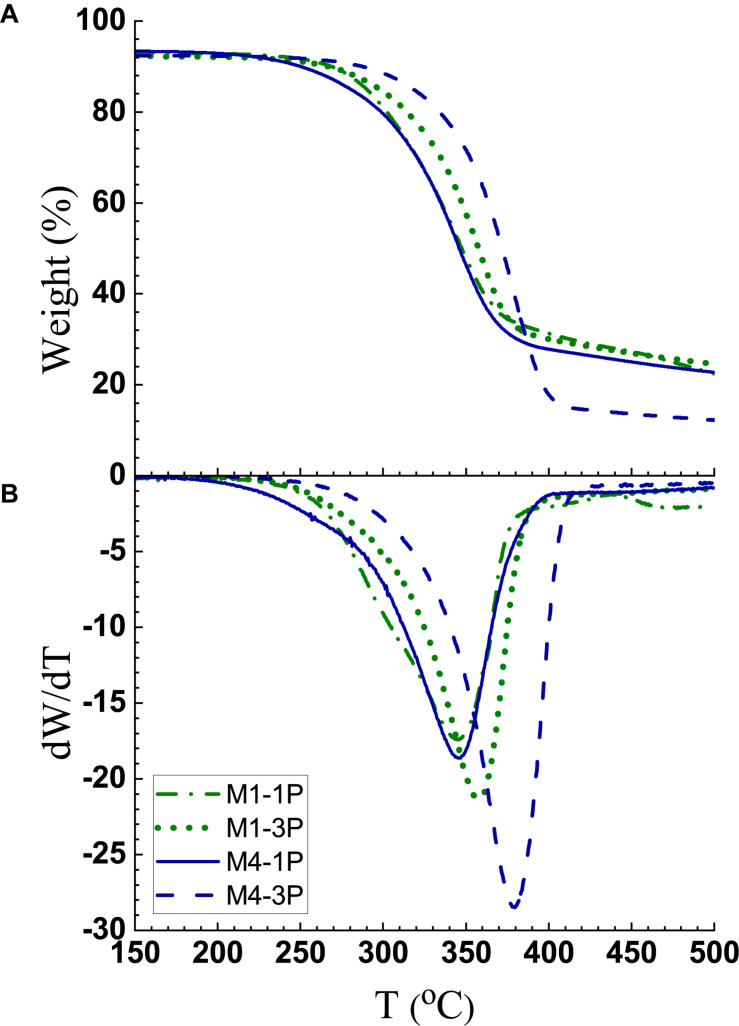
Thermogravimetric analyses of CNW samples from M1 and M4 after 1 and 3 passes. **(A)** Weight loss; **(B)** Derivative weight loss.

Thermogravimetric analyses (TGA) indicate CNWs M1-1P and M1-3P have good thermal stability. The onset of degradation, as defined as *dW*/*dT* ≥ –1, was at approximately 247°C and 252°C, respectively, as shown in Figure 6. These values are lower than 301°C for MA-CNCs produced at M60T100t45 reported previously, but notably higher than 218°C for S-CNCs (Chen et al., 2016).

It is interesting to notice that increasing fibrillation from one pass to three passes through homogenization increased thermal stability for both M1 CNWs and M4 CNFs. This is perhaps due to the increased exposure of hydroxyl groups with more fibrillation. Homogenization is primarily a shearing action that fibrillates, rather than cuts, cellulose fibrils resulting in minimal cellulose depolymerization. This can be seen from the DP data listed in Table 3, as well as from the sample morphologies shown in AFM images in Figure 3 and Supplementary Figure S3. The temperature shift at maximal weight loss is due to increased fibrillation and, as shown in Figure 6B, was obvious for both CNMs from M1 and M4. Therefore, we confidently demonstrate that reduction in thermal stability due to depolymerization is low.

**TABLE 3:** Surface charge and carboxyl group content along with dynamic light scattering (DLS) measured size and degree of polymerization of CNM samples listed in Table 1.

**CNM Label**	**Zeta-potential (mV)**	**DLS size (nm)**	**Carboxyl content (mmol/g)**	**DP**
M1-1P	−33.5 ± 1.1	693 ± 42	0.250	203 ± 0
M1-3P	−44.5 ± 1.6	649 ± 33	0.270	178 ± 1
M2-CNC	−37.5 ± 0.5	629 ± 25	0.252	N/A *
M2-1P	−34.1 ± 1.6	712 ± 52	0.113	N/A *
M2-3P	−56.6 ± 0.6	678 ± 46	0.129	177 ± 3
M3-1P	−32.5 ± 1.0		0.011	372 ± 1
M3-3P	−48.4 ± 2.8		0.016	331 ± 2
M4-1P	−30.6 ± 3.6			683 ± 8
M4-3P	−30.0 ± 1.8			629 ± 7
P1-1P	−30.7 ± 3.6			
P1-3P	−31.8 ± 2.9			
P3-1P	−27.4 ± 2.2			
P3-3P	−31.7 ± 2.6			

*Not analyzed due to limited sample available.

#### Surface Properties

Surface functionalization and charge are important properties to dispersion. M2-CNCs had more than double the carboxyl group content than the corresponding CNWs M2-1P and M2-3P (0.25, 0.11 and 0.13 mmol/g, respectively), as shown in Table 3 and in agreement with our earlier lab-scale study (Chen et al., 2016); and the higher-severity M1-CNWs had a greater carboxyl group content (≥ 0.25 mmol/g) than the M2-CNWs, as expected.

M1-1P and M2-1P had surface charge similar level to M2-CNC (−33.5, −34.1, and −37.5 mV, respectively). Increasing mechanical fibrillation increased surface charge for both M1-3P and M2-3P to approximately −45 mV or higher. These numbers suggest that CNWs from M1 and M2 through mechanical fibrillation are just as easily dispersible as M2-CNC.

Further reduction of hydrolysis severity decreased carboxylation as expected (Chen et al., 2016), as can be seen from M3 and M4 samples. Notably, however, despite the very low carboxyl content of only approximately 0.01 mmol/g, surface charge exceeds −30 mV, which is sufficient for dispersion. This suggests that surface charge is affected not only by carboxyl groups but also by hydroxyl groups. With three passes through homogenization, surface charge was increased to over −45 mV for both M2-3P and M3-3P.

Similarly, LCNF samples are also charged, but lower than the MA-produced CNM samples (Table 3). This is because p-TsOH does not functionalize fibers. Surface charges of LCNFs from P1 and P3 treatments were below −30 mV. As was the case before, surface charge increased with more fibrillation.

### Aqueous Suspension Rheology

**FIGURE 7:**
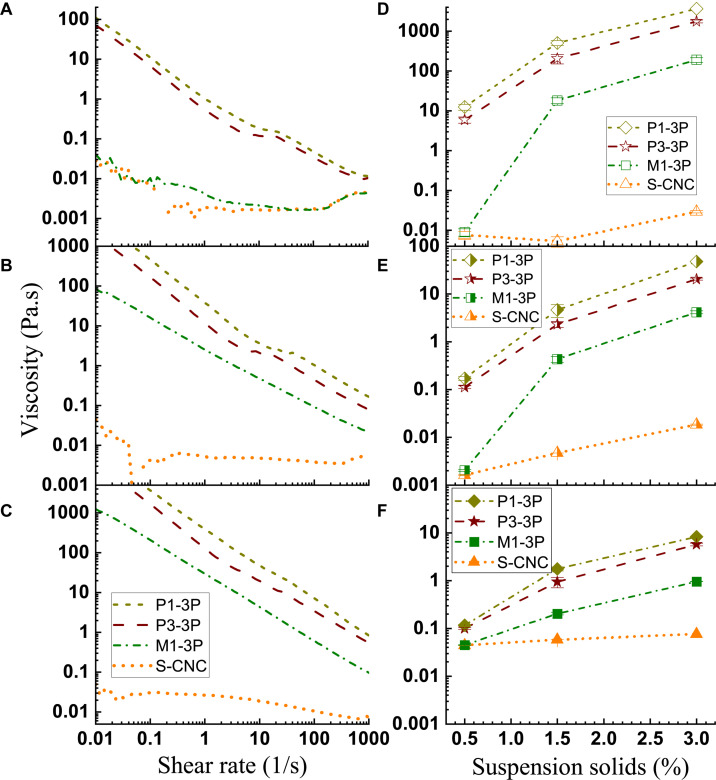
Effects of shear rate and CNM solids loading on CNM suspension viscosity. Left column: Solids loadings = 0.5 wt% **(A)**; 1.5 wt% **(B)**; 3.0 wt% **(C)**. Right column: Shear rate = 0.1 1/s **(D)**; 10 1/s **(E)**; 1000 1/s **(F)**.

As shown in Figures 7A–C, the S-CNC suspensions have typical semi-diluted CNC suspension rheology behavior up to 3 wt%, as also indicated in literature (Orts et al., 1998; de Souza Lima and Borsali, 2004; Oguzlu et al., 2017), i.e., shear thinning at low shear rates, a plateau region at moderate shear rates, then shear thinning again at high shear rates. CNW M1-3P at 0.5 wt% concentration (Figure 7A) also shows semi-diluted suspension behavior. As a matter of fact, its viscosity vs. shear rate curve almost overlaps with the curve for S-CNCs. However, at 1.5 wt%, M1-3P behaves similar to concentrated S-CNC suspension, as reported in the literature (Bercea and Navard, 2000; Shafiei-Sabet et al., 2012, 2014), i.e., viscosities decreased linearly with increased shear rate.

Compared with the S-CNCs with a mean aspect ratio of 7.7, CNW M1-3P has a greater aspect ratio of 12.2 (Supplementary Table S3). As a result, M1-3P has a greater range of viscosity within a very narrow concentration range under the same shear rate. For example, M1-3P viscosity increased over three orders of magnitude at a low shear rate of 0.1 1/s when suspension concentration was increased from 0.5 wt% to 1.5 wt% as shown in Figure 7D. There is essentially no change in viscosity for S-CNCs. At a moderate shear rate of 10 1/s, M1-3P viscosity increased approximately three orders of magnitude, much greater than the increase for S-CNCs when concentration was increased from 0.5 wt% to 1.5 wt% (Figure 7E). Even at a high shear rate of 1000 1/s, M1-3P viscosity increased approximately one order of magnitude but S-CNCs viscosity had negligible increase when concentration was increased from 0.5 wt% to 3 wt% (Figure 7F). This indicates that CNW M1-3P can be a much more effective rheology modifier than S-CNCs.

The two lignin-containing LCNMs showed some interesting rheological behavior. First, CNF P3-3P, which has a higher lignin content of 18.8% than the 9.6% of P1-3P (Table 1), has a lower viscosity under the same shear rate at the same suspension concentration, despite having much more entangled fibril networks (Supplementary Figure S3) than P1-3P (Figure 3). This indicates that lignin as a hydrophobic molecule plays a significant role in the rheological properties of aqueous lignin-containing CNF suspension, primarily due to limiting interactions with water. Second, it appears that both suspensions have a plateau region at suspension concentration below 1.5 wt% (Figures 7A,B). At 3 wt%, this plateau disappears (Figure 7C) and viscosity decreased linearly with shear rate, similar to concentrated CNC suspension. Due to their fibril network morphology (Figure 3 and Supplementary Figure S3), these two LCNM suspensions have relatively large viscosity ranges, as shown in Figures 7D–F. However, their ranges are smaller to the viscosity range of M1-3P except at very high shear rates (Figure 7F). Overall, the rheology properties of these two suspensions were very similar.

### Applications of CNWs and Final Thoughts

The CNWs from M2-3P (Figure 3) were used for producing unsaturated polyester resin (UPR) nanocomposite, though incorrectly labeled in the study (DiLoreto et al., 2019). It was demonstrated that freeze-dried M2-3P can be directly used to reinforce UPR and resulted in 61% and 48% increase in glassy and rubbery modulus, respectively, over the UPR baseline; and glass transition temperature was increased by 12°C. Using S-CNCs decreased UPR nanocomposite tensile strength. Equivalent gains in glassy and rubbery modulus were achieved only after modifying S-CNCs using methyl(triphenyl) phosphonium; however, this resulted in only a 4.5°C gain in glass transition temperature.

Based on the rheology data presented here, we see CNWs being particularly suitable for rheology modification and 3D printing. In this study, we demonstrated scale-up production with good control of sample morphology. Because MA is a solid acid with a relatively low solubility at room temperature, solidification to partially recover acid can be readily achieved (Chen et al., 2016; Cai et al., 2020a). Using mature technology, the remaining acid can be used to dehydrate the dissolved xylose to furfural at elevated temperatures (Cai et al., 2020a). When commercial bleached pulp fibers are used, the spent liquor is fairly clean after furfural dehydration and distillation and can be simply reused (Cai et al., 2020a). By producing CNWs with morphological and surface charge properties similar to those of CNCs but without dialysis, we can substantially reduce water usage to achieve sustainable production with favorable yields.

## Conclusion

This study describes a class of cellulosic nanomaterials produced at pilot-scale using maleic acid, an easily recyclable solid dicarboxylic acid and FDA approved indirect food additive per code of federal regulation 21CFR175-177, hydrolysis with subsequent mechanical nanoscale fibrillation, which we call cellulosic nanowhiskers (CNWs). Although crystallinity is not required in classifying CNWs, we characterized crystallinity, along with morphology, surface properties, and suspension rheology. We demonstrated some applications in which CNWs outperform the more expensive crystalline S-CNCs. We clarified why CNCs should not be used for applications that do not require crystalline property, such as rheology modifiers and 3D printings. By both Raman spectroscopy and X-Ray diffraction measurements, we demonstrated the reduction in crystallinity of CNMs with mechanical fibrillation. We therefore put forth the argument that CNWs should not be termed CNCs, although CNWs such as those from MA hydrolysis, can be crystalline with very similar morphology and surface charge to CNCs. Furthermore, we demonstrated that the surface carboxylated CNWs produced from maleic acid hydrolysis have good surface charge of −35 mV or higher that facilitated dispersion suitable for a variety of applications to substitute CNCs. Both CNW and lignin-containing LCNW suspensions, within a very narrow concentration range, exhibit a wide range of viscosity 0.001 to 1000 Pa.s under a range of shear rates for better performance as a rheological modifier compared to the viscosity of 0.001 – 0.1 Pa.s of traditional sulfuric acid S-CNCs. Finally, the scale-up showed good control of product morphology.

## Data Availability Statement

All datasets presented in this study are included in the article/Supplementary Material.

## Author Contributions

HW conducted CNM characterization including AFM, carboxyl group and surface charge, and CNM suspension rheology. JJZ conducted post-fibrillation enzymatic treatment and AFM of the treated samples. QM conducted fractionation studies to develop scale-up factors. UA conducted Raman scattering characterization and crystallinity analyses. RG and RR conducted pilot-scale CNMs and S-CNC production. CB conducted x-ray crystallinity measurements. JYZ initiated the research and wrote most of the manuscript. All authors contributed to the article and approved the submitted version.

## Conflict of Interest

Zhu and RG are co-inventors of the dicarboxylic acid hydrolysis process for producing CNMs. The remaining authors declare that the research was conducted in the absence of any commercial or financial relationships that could be construed as a potential conflict of interest.
