# Supplementary material for: Pilot-Scale Production of Cellulosic Nanowhiskers With Similar Morphology to Cellulose Nanocrystals
Source: Front Bioeng Biotechnol. 2020 Sep 4;8:565084. doi: 10.3389/fbioe.2020.565084 (PMC7500145; doi:10.3389/fbioe.2020.565084)
Supplement: Supplementary file 1 [file Data_Sheet_1.docx]

Supplementary Material

1. **Combined Hydrolysis Factor (CHF) and Combined Delignification Factor (CDF)**

In our earlier a series of studies, we developed a combined hydrolysis factor for predicting cellulose polymerization CHF_G_ (subscript G stands for glucan or cellulose) and CHF_X_ (subscript X stands for xylan) for predicting xylan dissolution for bleached pulp fibers. We also developed a combined delignification factor (CDF) for predicting delignification.

${CHF}_{G}=\exp\left( \alpha-\frac{E}{RT}+\beta C^{\varepsilon} \right)C\cdot t$ (S1a)

$\frac{DP}{{DP}_{BEP}}=F_{DP}e^{-j\cdot{CHF}_{G}}+S_{DP}e^{-{CHF}_{G}}+(1-F_{DP}-S_{DP})$ (S1b)

${CHF}_{X}=\exp\left( \alpha-\frac{E}{RT}+\beta C \right)C\cdot t$ (S1c)

$X_{R}=\left( 1-\theta\right)e^{-{CHF}_{X}}+\theta e^{-f\cdot{CHF}_{X}}$ (S1d)

$CDF=\exp\left( \alpha'-\frac{E^{'}}{RT}+\beta'C \right)C\cdot t$ (S2a)

$L_{\text{R}}=\left( 1-\theta'-{\theta'}_{\text{R}} \right)e^{-\text{CDF}}+\theta'\cdot e^{-f'\cdot\text{CDF}}+{\theta'}_{\text{R}}$ (S2b)

where *C* is MA or *p*-TsOH molar concentrations (mol/L), *R* = 8.314 (J/mol/K) is the universal gas content, *t* is reaction time in minutes, and *T* is reaction temperature in Kelvins. *α*, *α'*, and *β*, *β'* are adjustable parameters, *ε* is an exponential index, *E* and *E'* are apparent activation energy (J/mol). ${DP}_{BEP}$= 1027 is the degree of polymerization (DP) of feed BEP fibers, $F_{DP}$ and $S_{DP}$ are the respective fraction of cellulose depolymerization contribution from fast and slow reaction cellulose, *j* is the ratio of the reaction rates between rapid and slow depolymerizing cellulose. *X_R_* and *L_R_* are the percentage of xylan (CHF_X_ refers to CHF for xylan hydrolysis) and lignin retained on hydrolyzed water insoluble solids, respectively. *θ'* is the initial fraction of slow-reacting lignin. *f'* is the ratios of reaction rates between slow- and fast-reacting lignin. The concept of level off cellulose DP (LODP) is well known (Battista et al., 1956). Here $LODP=(1-F_{DP}-S_{DP})$. The measured DPs of MA-hydrolyzed BEP samples were previously fitted using Eqs. (S1a) and (S1b) to obtain ε, $F_{DP}$, $S_{DP}$ (Fig. S1), and *j* (Table S1). Similar fitting of experimental data of birch MDF delignification (Table S3) using *p*-TsOH was carried out (Fig. S2) to obtain fitting parameters in Eqs. (S2a) and (S2b) as listed in Table S1.

**References**

Wang, R., Chen, L., Zhu, J.Y., Yang, R. 2017. Tailored and integrated production of carboxylated cellulose nanocrystals (CNC) with nanofibrils (CNF) through maleic acid hydrolysis. *ChemNanoMat*, **3**(5), 328-335.

Zhu, W., Houtman, C.J., Zhu, J.Y., Gleisner, R., Chen, K.F. 2012. Quantitative predictions of bioconversion of aspen by dilute acid and SPORL pretreatments using a unified combined hydrolysis factor (CHF). *Process Biochem.*, **47**, 785-791.

Zhu, J., Chen, L., Gleisner, R., Zhu, J.Y. 2019. Co-production of bioethanol and furfural from poplar wood via low temperature (≤90 °C) acid hydrotropic fractionation (AHF). *Fuel*, **254**,115572 DOI:10.1016/j.fuel.2019.05.155.

1. **Supplementary Figures and Tables**

2.1 Supplementary Figures

Fig. S1 Xylan (A) dissolution and DP reduction (B) of BEP fibers through concentrated maleic acid hydrolysis fitted with kinetic-based reaction severities, combined hydrolysis factor (CHF). From (Wang et al., 2017 *ChemNanoMat*, **3**(5), 328-335)

Fig. S2 Xylan (A) and lignin (B) dissolution of birch MDF through hydrotropic fractionation using aqueous *p*-TsOH solution fitted with kinetic-based reaction severities, combined hydrolysis factor (CHF), and combined delignification factor, respectively.

| **1 pass** | **3 passes** |
| --- | --- |
| 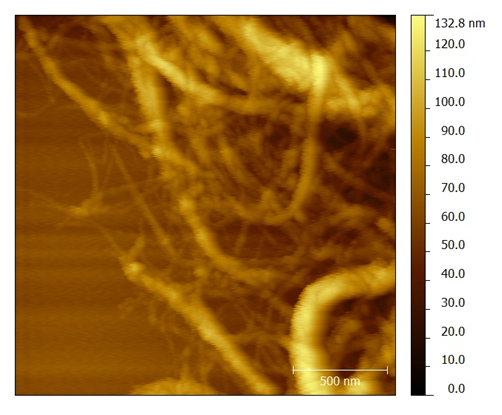 | 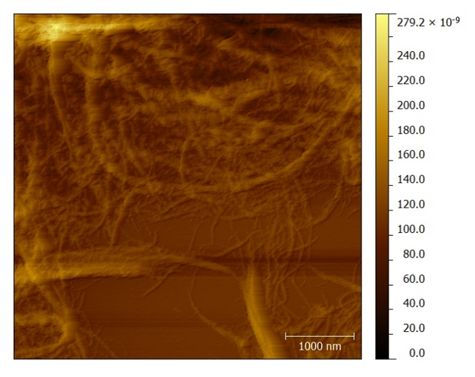 |
| 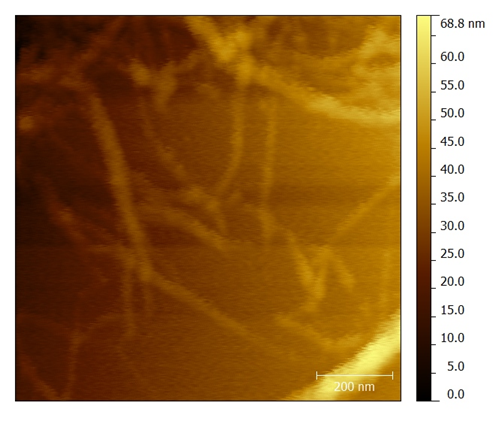 | 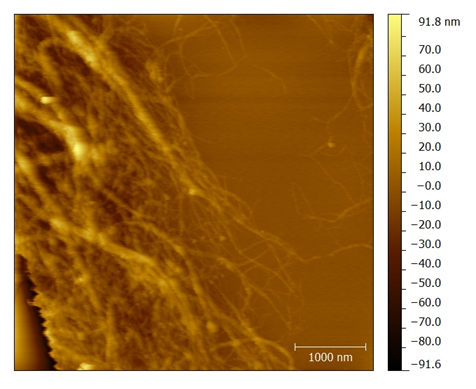 |

Fig. S3 Morphologies of CNFs (top row) from BEP under M40T80t60 (M4) and LCNFs (bottom row) MDF under P40T80t60 (P3), respectively.

Fig. S4 Temperature profile for run P2 (P50T81t27).

Fig. S5 Thermogravimetric analyses of LCNF samples P1 and P3 after 1 and 3 passes.

2.2 Supplementary Tables

Table S1 Fitting parameters for cellulose depolymerization of BEP fibers using concentrated maleic acid hydrolysis (Eqs. (1a) and (1b)) and for delignification of birch MDF fibers by concentrated *p*-TsOH fractionation (Eqs. (2a) and (2b)).

|  | DP (Eq. S1) ^1^ | Lignin (Eq. S2) | Unit |
| --- | --- | --- | --- |
| *E, E’* | 143,000 | 95,800 | J/mole |
| *α, α’* | 39.53 | 29.16 | none |
| *β, β’* | 0.3725 | 1.0833 | l/mole |
| *f, f’* | 0.0176 | 0.033 | none |
| *ε* | 0.5 |  | none |
| *F_DP_, θ’* | 0.467 | 0.343 | none |
| *S_DP,_* ${\theta'}_{R}$ | 0.328 | 0.2 | none |
| *j* | 58 |  | none |

^1^ Data from (Wang et al., 2017)

Table S2 Estimated CNW mean lengths and diameters in comparison with those of CNCs (based AFM shown in Fig. 3)

| **Samples** | **Mean Length (nm)** | **Mean Diameter (nm)** | **Aspect Ratio** |
| --- | --- | --- | --- |
| M1-1P | 400 | 38 | 10.5 |
| M1-3P | 365 | 30 | 12.2 |
| S-CNC | 185 | 24 | 7.7 |
| M2-1P | 325 | 28 | 11.6 |
| M2-3P | 270 | 22 | 12.3 |
| M2-CNC | 240 | 26 | 9.2 |
| P1-1P | 430 | 40 | 10.8 |
| Wood LCNC | 125 | 9 | 13.9 |

Table S3 Chemical compositions of *p*-TsOH fractionated birch samples under different treatment conditions. The numbers in the parentheses are component yields based on component in the untreated birch.

| **Sample Label ^1^** | **Water-insoluble solids (WIS)** | | | | |  |  |  |
| --- | --- | --- | --- | --- | --- | --- | --- | --- |
|  | Solids yield (%) | Glucan  (%) | Xylan  (%) | Mannan  (%) | Lignin  (%) | Glucose  (g/L) | Xylose  (g/L) |  |
| Untreated birch | 100 | 35.3 | 20.5 | 1.9 | 23.5 |  |  | |
| P25T60t30 | 82.0 | 37.7 (87.6) | 18.3 (73.1) | 1.6 (68.4) | 20.3 (70.8) | 0.01 | 0.02 | |
| P40T60t20 | 78.8 | 39.3 (87.7) | 17.0 (65.1) | 1.7 (70.8) | 20.6 (69.1) | 0.01 | 0.02 | |
| P40T60t60 | 73.5 | 40.3 (83.9) | 16.7 (59.8) | 1.5 (60.1) | 20.2 (63.1) | 0.01 | 0.05 | |
| P40T80t55 | 60.6 | 51.7 (88.7) | 13.3 (39.4) | 2.3 (73.3) | 17.6 (45.5) | 0.01 | 0.14 | |
| P50T80t20 | 56.7 | 59.2 (95.0) | 14.9 (41.4) | 2.4 (70.5) | 16.0 (38.5) | 0.03 | 2.92 | |
| P50T80t40 | 59.6 | 53.7 (90.6) | 13.0 (37.9) | 2.0 (63.1) | 15.3 (38.8) | 0.04 | 3.41 | |
| P50T80t55 | 60.1 | 54.5 (92.7) | 12.4 (36.4) | 2.2 (69.8) | 15.6 (40.0) | 0.10 | 4.20 | |
| P50T80t80 | 56.5 | 61.1 (97.8) | 11.4 (31.4) | 2.4 (72.1) | 13.8 (33.3) | 0.07 | 3.73 | |
| P60T80t70 | 55.1 | 62.1 (96.9) | 10.3 (27.7) | 2.4 (68.2) | 12.1 (28.5) | 0.06 | 4.18 | |
| P65T80t20 | 54.2 | 62.0 (95.1) | 14.0 (36.9) | 2.6 (74.5) | 11.6 (26.9) | 0.16 | 4.51 | |
| P70T80t90 | 53.2 | 65.5 (98.7) | 10.8 (27.9) | 2.5 (70.9) | 8.7 (19.6) | 0.21 | 4.43 | |
| P70T80t120 | 54.2 | 63.7 (97.8) | 8.2 (21.5) | 2.1 (61.1) | 14.3 (33.1) | 0.02 | 2.26 | |
| P80T80t20 | 51.3 | 67.7 (98.4) | 12.2 (30.4) | 2.5 (67.9) | 7.2 (15.7) | 0.01 | 0.12 | |

^1^ (Pxx, Txx, txx) stands for *p*-TsOH concentration in wt%, reaction temperature in ºC and reaction duration in min.

^2^ Yields are based on xylan content in birch. HMF in spent liquors was not detectable.
